# Silicosis Mortality Trends and New Exposures to Respirable Crystalline Silica — United States, 2001–2010

**Published:** 2015-02-13

**Authors:** Ki Moon Bang, Jacek M. Mazurek, John M. Wood, Gretchen E. White, Scott A. Hendricks, Ainsley Weston

**Affiliations:** 1 Division of Respiratory Disease Studies, National Institute for Occupational Safety and Health, CDC; 2 Divison of Safety Research, National Institute for Occupational Safety and Health, CDC

Silicosis is a preventable occupational lung disease caused by the inhalation of respirable crystalline silica dust and can progress to respiratory failure and death (1). No effective specific treatment for silicosis is available; patients are provided supportive care, and some patients may be considered for lung transplantation. Chronic silicosis can develop or progress even after occupational exposure has ceased (1). The number of deaths from silicosis declined from 1,065 in 1968 to 165 in 2004 (2). Hazardous occupational exposures to silica dust have long been known to occur in a variety of industrial operations, including mining, quarrying, sandblasting, rock drilling, road construction, pottery making, stone masonry, and tunneling operations (1). Recently, hazardous silica exposures have been newly documented during hydraulic fracturing of gas and oil wells and during fabrication and installation of engineered stone countertops (3,4). To describe temporal trends in silicosis mortality in the United States, CDC analyzed annual multiple cause-of-death data for 2001–2010 for decedents aged ≥15 years.* During 2001–2010, a total of 1,437 decedents had silicosis coded as an underlying or contributing cause of death. The annual number of silicosis deaths declined from 164 (death rate† = 0.74 per 1 million population) in 2001 to 101 (0.39 per 1 million) in 2010 (p = 0.002). Because of new operations and tasks placing workers at risk for silicosis, efforts to limit workplace exposure to crystalline silica need to be maintained.

For this analysis, decedents for whom the *International Classification of Diseases, 10th Revision* code J62 (pneumoconiosis due to dust containing silica [silicosis]§) was assigned as either the underlying¶ or contributing cause of death were identified from 2001–2010 mortality data. Deaths of persons aged ≥15 years were analyzed. Trends in annual age-adjusted death rates per 1 million population were examined using a first-order autoregressive linear regression model. Differences in death rates were considered to be statistically significant if 95% confidence intervals did not overlap.

During 2001–2010, 1,437 decedents had silicosis coded as the underlying or contributing cause of death. Of these, 28 (1.9%) were aged 15–44 years, 1,370 (95.3%) were males, and 1,236 (86.0%) were whites (Table). The overall age-adjusted silicosis death rate for blacks (0.87 per 1 million) was significantly higher than the rate for whites (0.59) and other races (0.16). The age-adjusted silicosis death rate for males (1.39 per 1 million) was significantly higher than the rate for females. The annual number of silicosis deaths declined from 164 (0.74 per 1 million) in 2001 to 101 (0.39) in 2010 (p for trend = 0.002).

## Discussion

A statistically significant decline in silicosis death rates was observed during 2001–2010. However, silicosis deaths still occurred among persons aged 15–44 years. Of 28 decedents aged 15–44 years, the youngest was aged 19 years. This would be consistent with the decedent developing acute silicosis after an extremely high exposure to respirable crystalline silica. Such findings indicate the importance of educating at-risk workers and their employers regarding the dangers of exposure to respirable crystalline silica in the workplace. The disparities by sex and by race reflect differences in the composition of the workforces in the industries and occupations placing workers at risk for exposure to crystalline silica dust.**

Approximately 2 million U.S. workers remain potentially exposed to respirable crystalline silica (5). Occupational exposures to dust containing crystalline silica have long been known to occur in mining, quarrying, sandblasting, pottery making, rock drilling, road construction, stone masonry, and tunneling operations (1,5). Despite enforceable limits†† on worker exposure to respirable crystalline silica, substantial overexposures continue to occur in the United States (3)*.* Moreover, new job tasks that place workers at risk for silicosis continue to emerge.

In 2004, occupational disease surveillance programs in Michigan, New Jersey, Massachusetts, New York, and Ohio reported nine confirmed cases of silicosis among technicians who performed sandblasting in dental laboratories (6); in 2013, there were approximately 37,000 dental laboratory technicians in the United States.§§ In a 2012 report from Israel, a 2014 report from Spain, and a 2015 report from the United States, silicosis has been documented among workers exposed to respirable crystalline silica dust during the fabrication and installation of quartz-containing engineered stone products used primarily for kitchen and bathroom countertops (4,7,8). A 2013 report documented high levels of exposure to respirable crystalline silica during hydraulic fracturing of gas and oil wells (3). Moreover, a 2010 study reported an excess risk for silicosis in coal miners that was associated with silica as a component of coal mine dust formed during drilling, crushing, and loading of mine material (9). In 2013, there were approximately 204,000 oil and gas extraction industry workers and approximately 80,000 coal mining industry workers in the United States.¶¶ Finally, although not in the United States, silicosis cases have been reported in other occupational settings, including among denim sandblasters (10).

**What is already known on this topic?**

Silicosis is an occupational lung disease caused by inhalation of respirable crystalline silica in a variety of industrial operations, including mining, quarrying, road construction, masonry, and tunneling. From 1968 to 2004, silicosis deaths in the United States declined from 1,065 per year to 165.

**What is added by this report?**

Although silicosis deaths decreased significantly from 164 in 2001 to 101 in 2010, they continued to occur among young persons, with 28 deaths reported among persons aged 15–44 years during 2001–2010. New work tasks, including hydraulic fracturing, sandblasting denim, and engineered stone countertop fabrication and installation, can lead to overexposure to respirable crystalline silica.

**What are the implications for public health practice?**

Because of the serious health and socioeconomic consequences of silicosis, new operations and tasks placing workers at risk for silicosis, and the continuing occurrence of silicosis deaths, efforts to limit workplace exposure to crystalline silica need to be maintained. In addition, the long latency of silicosis warrants continuing surveillance. The Occupational Safety and Health Administration and CDC recommend best practices for protecting workers, including the use of engineering controls and respiratory protection.

In 1999, the Council of State and Territorial Epidemiologists made silicosis a nationally notifiable condition.*** In addition, because current permissible exposure limits for respirable crystalline silica do not adequately protect workers, the Occupational Safety and Health Administration (OSHA) has proposed amending the current standards. One of the proposed changes is a lower permissible exposure limit (5).

The findings in this report are subject to at least three limitations. First, silicosis deaths were not validated by medical records or follow-up with health care providers, thus findings might be subject to misclassification. Second, no individual work history is reported on death certificates. Therefore, it was not possible to identify those industries and occupations where the decedents’ exposures to crystalline silica occurred. Finally, inhalation of respirable crystalline silica can cause diseases other than silicosis, such as lung cancer and chronic obstructive pulmonary disease (1,5), which are not considered in this analysis.

Effective silicosis prevention strategies for employers recommended by OSHA††† and CDC’s National Institute for Occupational Safety and Health§§§ are available. Comprehensive silicosis prevention programs include substituting less hazardous noncrystalline silica alternatives when possible, implementing engineering controls (e.g., blasting cabinets, local exhaust ventilation, not using compressed air for cleaning surfaces, using water sprays to control airborne dust, and using surface wetting to prevent dust from becoming airborne when cutting, drilling, grinding, etc.), administrative and work practice controls, personal respiratory protective equipment, medical monitoring of exposed workers, and worker training. Because of the serious health and socioeconomic consequences of silicosis, new operations and tasks placing workers at risk for silicosis, and the continuing occurrence of silicosis deaths among young workers, effective primary prevention through elimination of exposure to respirable crystalline silica is critical. At the same time, because of the sometimes long latency of silicosis, with cases diagnosed years after exposure and often in retirement, ongoing silicosis surveillance is needed to track its prevalence in the United States.

## Figures and Tables

**TABLE:** Number and rate* of silicosis deaths, by selected characteristics and year — United States, 2001–2010

Characteristic	Age group (yrs)						Overall		

	15–44			≥45					
		
	No.	Rate	(95% CI)	No.	Rate	(95% CI)	No.	Rate	(95% CI)
**Total**	**28**	**0.01**	**(0.01–0.01)**	**1,409**	**0.58**	**(0.55–0.61)**	**1,437**	**0.59**	**(0.56–0.62)**
**Sex**									
Male	23	0.02	(0.01–0.03)	1,347	1.37	(1.30–1.44)	1,370	1.39	(1.32–1.46)
Female	5	0.00	—	62	0.04	(0.03–0.05)	67	0.05	(0.04–0.06)
**Race**									
White	22	0.01	(0.01–0.02)	1,214	0.57	(0.54–0.60)	1,236	0.59	(0.56–0.62)
Black	5	0.01	(0.01–0.05)	181	0.85	(0.72–0.98)	186	0.87	(0.74–1.00)
Other	1	0.01	(0.00–0.06)	14	0.15	(0.08–0.25)	15	0.16	(0.09–0.26)
**Year**									
2001	1	0.00	—	163	0.74	(0.63–0.85)	164	0.74	(0.63–0.85)
2002	5	0.02	(0.01–0.05)	143	0.64	(0.54–0.74)	148	0.66	(0.55–0.77)
2003	6	0.02	(0.01–0.07)	173	0.76	(0.65–0.87)	179	0.78	(0.67–0.89)
2004	3	0.01	(0.00–0.03)	163	0.70	(0.59–0.81)	166	0.71	(0.60–0.82)
2005	2	0.01	(0.00–0.04)	159	0.67	(0.57–0.77)	161	0.68	(0.57–0.79)
2006	6	0.02	(0.01–0.07)	120	0.49	(0.40–0.58)	126	0.52	(0.43–0.61)
2007	1	0.00	—	122	0.49	(0.40–0.58)	123	0.50	(0.41–0.59)
2008	2	0.01	(0.00–0.04)	146	0.58	(0.49–0.67)	148	0.58	(0.49–0.67)
2009	1	0.00	—	120	0.47	(0.39–0.55)	121	0.48	(0.39–0.57)
2010	1	0.01	(0.00–0.06)	100	0.38	(0.30–0.46)	101	0.39	(0.31–0.47)
p-value†	—§	—§		0.012	0.002		0.010	0.002	

**Abbreviation:** CI = confidence interval.

* Rate per 1 million persons, age-adjusted to the 2000 U.S. standard population.

† For 2001–2010 trend.

§ Trend test not performed because of small number of deaths.

## Footnotes

* Additional information available at http://webappa.cdc.gov/ords/norms.html.

† Death rates were age-adjusted to the 2000 standard U.S. population. The age intervals used were 15–34, 35–44, 45–54, 55–64, 65–74, 75–84, and ≥85 years.

§ Classic (chronic) silicosis results from exposure to respirable crystalline silica for >10 years; exposure to higher concentrations of silica for 5–10 years can cause accelerated silicosis, and symptoms of acute silicosis can sometimes develop within weeks of initial exposure to extremely high concentrations of silica.

¶ Underlying cause of death is defined as “the disease or injury which initiated the train of morbid events leading directly to death, or the circumstances of the accident or violence which produced the fatal injury.”

** Additional information available at http://www.bls.gov/cps/wlf-databook-2013.pdf.

†† Additional information available in “Lowering Miners’ Exposure to Respirable Coal Mine Dust, Including Continuous Personal Dust Monitors; Final Rule” (http://www.gpo.gov/fdsys/pkg/FR-2014-05-01/pdf/2014-09084.pdf) and “Criteria for a Recommended Standard: Occupational Exposure to Respirable Coal Mine Dust” (http://www.cdc.gov/niosh/docs/95-106/pdfs/95-106.pdf).

§§ Additional information available at http://www.bls.gov/oes/current/oes_nat.htm.

¶¶ Additional information available at http://www.bls.gov/opub/ee/2014/ces/table1a_201401.pdf.

*** Available at http://c.ymcdn.com/sites/www.cste.org/resource/resmgr/ps/09-oh-01.pdf.

††† Available at https://www.osha.gov/pls/oshaweb/owadisp.show_document?p_table=standards&p_id=12716.

§§§ Available at http://www.cdc.gov/niosh/topics/silica.

## References

[1] National Institute for Occupational Safety and Health (2002). NIOSH hazard review Health effects of occupational exposure to respirable crystalline silica.

[2] Mazurek JM, Attfield MD (2008). Silicosis mortality among young adults in the United States, 1968–2004. Am J Ind Med.

[3] Esswein EJ, Breitenstein M, Snawder J, Kiefer M, Sieber WK (2013). Occupational exposure to respirable crystalline silica during hydraulic fracturing. J Occup Environ Hyg.

[4] Kramer MR, Blanc PD, Fireman E (2012). Artificial stone silicosis: disease resurgence among artificial stone workers. Chest.

[5] Occupational Safety and Health Administration, US Department of Labor Occupational exposure to respirable crystalline silica; proposed rule.

[6] CDC (2004). Silicosis in dental laboratory technicians—five states, 1994–2000. MMWR Morb Mortal Wkly Rep.

[7] Friedman GK, Harrison R, Bojes H, Worthington K, Filios M (2015). Notes from the field: silicosis in a countertop fabricator—Texas, 2014. MMWR Morb Mortal Wkly Rep.

[8] Pérez-Alonso A, Córdoba-Doña JA, Millares-Lorenzo JL, Figueroa-Murillo E, García-Vadillo C, Romero-Morillos J (2014). Outbreak of silicosis in Spanish quartz conglomerate workers. Int J Occup Environ Health.

[9] Laney AS, Petsonk EL, Attfield MD (2010). Pneumoconiosis among underground bituminous coal miners in the United States: is silicosis becoming more frequent?. Occup Environ Med.

[10] Bakan ND, Özkan G, Çamsari G (2011). Silicosis in denim sandblasters. Chest.

